# Ecological Interactions on Sandy Beach Ecosystems: A Global Synthesis of Mole Crabs and New Insights into *Emerita brasiliensis* and *Emerita rathbunae* (Crustacea, Decapoda, Anomura, Hippidae)

**DOI:** 10.3390/biology15040311

**Published:** 2026-02-10

**Authors:** Rayane Romão Saad Abude, Michel E. Hendrickx, José Salgado-Barragán, Mayra I. Grano-Maldonado, Martín García-Varela, Alvaro Esteves Migotto, Joel Campos de Paula, Matheus Augusto, Daniel Andrade Moreira, Thiago Estevam Parente, Gisele Lôbo-Hajdu, Tatiana Medeiros Barbosa Cabrini

**Affiliations:** 1 Post-Graduate Program in Ecology and Evolution, Institute of Biology Roberto Alcantara Gomes, Universidade do Estado do Rio de Janeiro (UERJ), Rio de Janeiro 22290-255, Brazil; 2 Laboratory of Marine Ecology, Department of Ecology and Marine Resources, Institute of Biosciences, Universidade Federal do Estado do Rio de Janeiro (UNIRIO), Rio de Janeiro 22290-255, Braziltatiana.cabrini@unirio.br (T.M.B.C.); 3 Laboratory of Benthic Invertebrates, Mazatlán Unit, Institute of Marine Sciences and Limnology, Universidad Nacional Autónoma de Mexico (UNAM), Sinaloa 82047, Mexico; 4 Facultad de Ciencias del Mar, Universidad Autónoma de Sinaloa (UAS), Sinaloa 82000, Mexico; 5 Department of Zoology, Institute of Biology, Universidad Nacional Autónoma de Mexico (UNAM), Ciudad Universitaria, Mexico City 04510, Mexico; 6 Center for Marine Biology, Universidade de São Paulo (USP), São Paulo 11612-109, Brazil; 7 Laboratory of Algal Biology and Taxonomy, Universidade Federal do Estado do Rio de Janeiro (UNIRIO), Rio de Janeiro 22290-255, Brazil; 8 Laboratory of Applied Genomics and Bioinnovations, Oswaldo Cruz Institute (IOC), Fundação Oswaldo Cruz (Fiocruz), Rio de Janeiro 21040-360, Brazil; 9 Marine Genetics Laboratory, Department of Genetics, Institute of Biology, Universidade do Estado do Rio de Janeiro (UERJ), Rio de Janeiro 20550-900, Brazil

**Keywords:** parasitism, epibiosis, predation, algae, invertebrates

## Abstract

Sandy beaches are dynamic intertidal ecosystems where ecological interactions play a critical yet often overlooked role in shaping community structure and population dynamics. This study presents a global synthesis of ecological interactions involving mole crabs of the genus *Emerita* (Crustacea: Decapoda: Hippidae), complemented by new field and laboratory findings. Through a literature review and targeted sampling, we documented multiple interaction types, including predation, parasitism, epibiosis, competition, and symbiosis, highlighting their ecological and potential evolutionary implications. Predation and parasitism were the most frequently reported interactions worldwide. Our new empirical observations revealed, for the first time, the association of *Eucheilota* (Hydrozoa) and *Maritrema* sp. (Digenea) with *E. rathbunae*, as well as annual infection patterns by *Profilicollis altmani* (Acanthocephala) and algal epibiosis in *E. brasiliensis*. These interactions influence key biological processes such as burrowing, reproduction, and survival, ultimately affecting species distribution and population structure. Overall, our findings reinforce the central role of ecological interactions in the functioning and conservation of sandy beach ecosystems, particularly under growing anthropogenic pressures.

## Simple Summary

Sandy beaches are dynamic environments where many animals interact, shaping how they live, grow, and survive. Mole crabs of the genus *Emerita* are small crustaceans that live buried in the sand, and they take part in a surprising variety of interactions with other organisms. In this study, we gathered information from scientific publications worldwide and added new observations from beaches along the Atlantic and Pacific coasts of the Americas. We found that these crabs are commonly preyed on and parasitized, and they can also host algae and other small organisms on their bodies. Some of these interactions had never been recorded before, such as the association of certain hydrozoans and parasites with these crabs. These relationships can change how the crabs feed, move, and reproduce and may even alter where they are found along the coast. Understanding these interactions underscores the importance of mole crabs to sandy beach ecosystems and the need to protect these habitats, especially as human activities increasingly impact coastal areas.

## 1. Introduction

Ecological interactions represent the relationships formed between individuals sharing resources, space, and habitat [1]. An ecological interaction is initiated when a physical association is established between individuals, as observed in predation, competition, mutualism, or parasitism. In each scenario, reciprocal influences on the involved species are present [2]. Such interactions are seldom reported in beach environments, and their ecological effects remain inadequately understood.

Nevertheless, these complex ecosystem processes emerge from microscale interactions and determine nutrient cycling, food webs, and species life histories [3]. In microscale processes, interactions can cascade, impacting higher levels of biological organization (populations and communities) [4]. Deleterious effects of interactions through parasitism and epibiosis have been reported for some sandy beach species [5,6]. However, ecological interactions can also represent evolutionary advantages at both the individual and population levels [7].

Sandy beaches form complex systems that support specialized and diverse organisms, where marine, intertidal, and terrestrial species interact [8,9]. While physical factors mainly control exposed beaches, ecological interactions are more visible in protected environments [10,11]. Across the environmental gradient, these biotic interactions remain fundamental, dictating individual behavior and survival and shaping population dynamics [11,12].

Among the taxa in these environments is the genus *Emerita* (Crustacea: Decapoda: Anomura: Hippidae), which is broadly distributed in the swash zone of sandy beaches on the Pacific, Atlantic, and Indian Oceans [13,14]. During their benthic phase, individuals range from 2 mm to 4 cm in cephalothorax length [15,16]. *Emerita* species exhibit rapid burial in sediment during the swash and are suspension feeders, capturing suspended particulate organic matter from the water column using their setose antennules when submerged [16,17]. As trophic intermediaries, they are a key component of their ecosystem’s food web.

*Emerita brasiliensis* inhabits sandy beaches along the South Atlantic coast of Argentina, Brazil, and Uruguay, while *E. rathbunae* is present on tropical and subtropical beaches of the Eastern Pacific in México, Costa Rica, Colombia, Ecuador (including the Galapagos), and Peru [14,18,19]. Understanding these interactions is key to deciphering species’ life modes, especially as sandy beaches face anthropogenic changes. Since interactions regulate populations, it is urgent to understand their dynamics in *Emerita*, which is now experiencing global declines [14].

In this context, the present study aims to (1) provide a systematic synthesis of documented ecological interactions involving *Emerita,* using an extensive literature review, and (2) present original empirical data regarding the ecological associations of *E. brasiliensis* and *E. rathbunae* from the Atlantic coast of Brazil and the Pacific coast of Mexico, respectively. By integrating historical knowledge with novel field-based findings, we aim to provide a broader perspective on the ecological role of these decapod crustaceans across distinct geographical regions.

## 2. Global Synthesis of Ecological Interactions in *Emerita*

### 2.1. Methodological Approach for Literature Review

To characterize the diversity and nature of ecological interactions within *Emerita*, we conducted a comprehensive literature search in Google Scholar from January to November 2025, with no date restrictions, thereby considering all available historical records. The review was designed to address the following: which ecological interactions involving *Emerita* have been documented, and how these interactions are categorized by type.

The search strategy employed combinations of taxonomic and common names (‘*Emerita*’, ‘Hippidae’, ‘mole crab’, ‘sand crab’, and ‘sand flea’) with interaction-specific keywords (‘interactions’, ‘relationships’, ‘parasitism’, ‘epibiosis’, ‘symbiosis’, ‘mutualism’, ‘commensalism’, ‘competition’, ‘predation’, ‘diet’, and ‘mating’ (e.g., “*Emerita”* and “parasitism”; “*Emerita*” and “competition”). Both peer-reviewed scientific articles and gray literature were considered. The inclusion criteria prioritized any study documenting ecological interactions involving the genus *Emerita*, regardless of whether it was the primary focus of the research. This allowed the inclusion of diverse sources. The screening workflow consisted of (i) an initial screening of titles and abstracts to identify potential ecological records; (ii) a full-text review to confirm the nature of the interaction; and (iii) data extraction from the selected studies.

**Figure 1:**
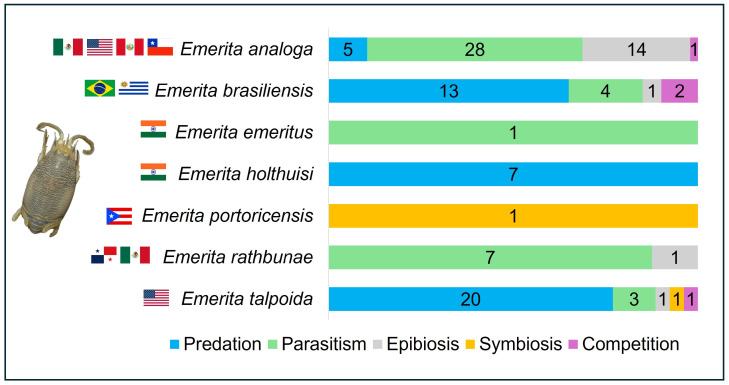
Global records of ecological interactions in *Emerita* species: *E. analoga* (Mexico, USA, Peru, Chile); *E. brasiliensis* (Brazil, Uruguay); *E. emeritus* and *E. holthuisi* (India); *E. portoricensis* (Puerto Rico); *E. rathbunae* (Panama, Mexico); *E. talpoida* (USA).

After screening for alignment with the study’s scope, 58 publications reporting ecological interactions in *Emerita* were retained for synthesis. Data from these studies were categorized by species, interaction type, and geographic location. The frequency of interactions was defined as the total number of unique scientific publications that identified and described a particular type of ecological relationship (e.g., parasitism, predation, or commensalism) involving *Emerita* species. The resulting dataset included seven species of *Emerita* (Figure 1) and 55 interacting species distributed across 43 genera, as summarized in Table 1 and Table S1.

### 2.2. Synthesis of the Global Literature Review

#### 2.2.1. Predation

Predation was the most documented interaction in our review, reported 45 times in the studies analyzed. These records span four species (*E. talpoida*, *E. brasiliensis*, *E. holthuisi*, and *E. analoga*) and involve a diverse predator assemblage including fishes, coastal birds, crustaceans, mollusks, and annelids. This highlights the ecological role of mole crabs in supporting various taxonomic groups within the food web, since they are filter feeders and nutrient cyclers.

**Figure 2:**
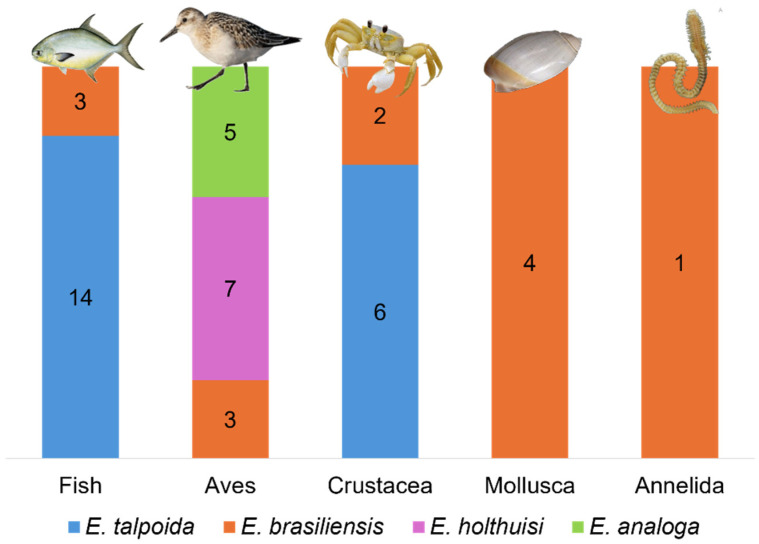
Global records of predator groups on *Emerita*.

Fishes are the most frequently recorded predator group (Figure 2). Quantitative data in these studies are primarily derived from stomach content analyses. Fish species that prey on mole crabs belong to various taxonomic groups (Table 1) and are commonly found in coastal environments, sharing a common habit of foraging in the surf zone and shallow coastal waters where mole crabs are abundant [20,21,22,23,24,25]. Average body size among these fish species varies considerably, reflecting different predatory strategies and prey size preferences. To capture their prey, these fish typically rely on visual and tactile cues, often exhibiting benthic or near-bottom foraging behavior adapted to dynamic sandy environments [26,27].

Unlike fish, coastal birds (Aves) showed a seasonal preference for preying on mole crabs, with winter being the primary season during which some species—*Charadrius* spp., *Tringa nebularia*, *Numenius phaeopus*, *Arenaria interpres*, *Xenus cinereus*, and *Pluvialis squatarola*—intensified their predation on mole crabs on Indian beaches [28]. Depending on the annual cycle and life history of the predator species, predation during specific seasons may pose greater or lesser risks to the establishment of mole crab populations. *Calidris alba* (Charadriiformes: Scolopacidae), a small shorebird, likely prefers the recently recruited mole crabs. This is supported by observations of a reduction in juvenile abundance when *C. alba* was foraging [29]. The size of the bird also appears to influence its feeding preference for adult or juvenile prey [30].

Larger crustaceans, mollusks, and annelids inhabiting sandy beaches are also important predators of *Emerita* [31,32]. Since *Emerita* is a filter feeder, it serves as a trophic link in sandy beach ecosystems, particularly when these environments are considered closed systems. *Emerita brasiliensis* has been suggested as the main prey of the gastropod *Olivancillaria vesica* [33]. Predation on *Emerita* by members of the sandy beach macrofauna that occupy the top of their food web has been studied since the 1970s through field observations, rarely by stomach and gut content analyses, and, more recently, stable isotope analysis. Predators of *Emerita* belonging to the macrofauna encompass the brachyuran crabs *Arenaeus cribrarius*, *Ocypode quadrata*, and *Ovalipes* spp.; the gastropods *O. vesica* and *O.auricularia*; and the annelid *Hemipodus olivieri* [34,35,36,37,38,39,40]. Among macrofaunal predators, no reported or inferable relationships were found between body size or life stage and prey size.

Prey occurrence and availability are determined by habitat quality and nutrient levels, which are key determinants of predator presence [40]. Healthy environments support more complex ecosystems, which are characterized by greater species richness, abundance, and ecological interactions, as well as increased predator presence [1]. Then, individuals from healthier environments are more likely to be preyed upon; however, because of ecosystem balance, predation does not threaten the establishment of the population. Paradoxically, in this context, higher environmental quality demands greater individual adaptation strategies to escape, maintain, and survive under predation pressure [41,42,43,44].

More heavily impacted beaches, which are constantly subjected to anthropogenic pressures, support fewer complex ecosystems, thereby affecting predator presence [45]. However, this does not imply greater survival or a higher abundance of prey species, especially those also affected by human pressures, such as *Emerita*, which is recognized as a bioindicator of beach quality [46]. Additionally, on beaches where *Emerita* individuals are removed for bait and human consumption [14], this removal is likely to affect the entire ecosystem by partially depleting an essential ecological component that significantly sustains the food web.

#### 2.2.2. Parasitism

Parasites are characterized by their ability to alter the behavioral patterns, metabolic rates, reproduction capacity, physiology, and/or morphology of their hosts [47,48]. Prevalence and intensity are widely recognized metrics in parasitological studies, where prevalence refers to the proportion of infected hosts within a population, while intensity denotes the number of parasites per infected host [49].

**Figure 3:**
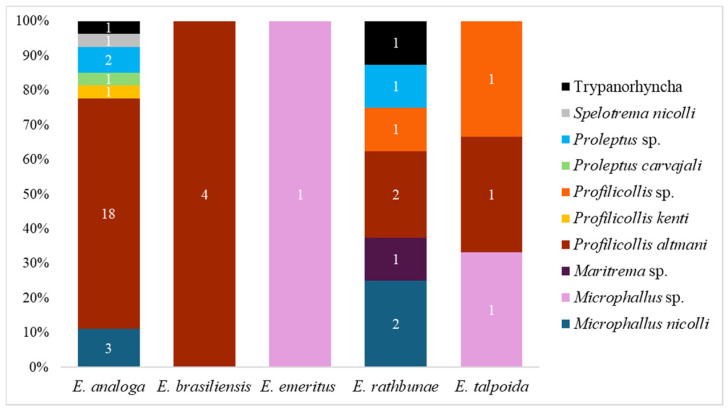
Global records of parasites in the *Emerita* species.

Our synthesis reveals that parasitism is a widely documented interaction for the genus, reported in 40 separate records across the reviewed literature. The highest concentration of these studies is focused on *E. analoga* (Figure 3). The parasitic fauna of *Emerita* includes acanthocephalans, platyhelminths, nematodes, and a facultative endoparasitic mollusk [50,51,52,53,54,55].

Quantitative insights from the literature indicate that acanthocephalans and platyhelminths are the dominant taxa. These parasites are typically heteroxenous, requiring intermediate hosts, such as the *Emerita* species, through which they reach their definitive hosts, usually coastal birds or fish. Parasites reproduce in the definitive hosts, releasing eggs into the environment via feces, which *Emerita* then ingest through filter feeding.

In *Emerita*, acanthocephalan infections can range from one to 22 individuals per host [55], whereas platyhelminth infections may reach up to 6000 individuals per host [12]. In both *taxa*, larger hosts tend to harbor higher parasite intensities [18,51]. Females of *Emerita* typically attain larger body sizes and longer lifespans than males [56], which likely explains the higher parasite prevalence and intensity observed in females [57]. Conversely, larger crabs have been observed to show a lower incidence of the platyhelminth *Microphallus nicolli*, possibly reflecting the selective removal of heavily infected individuals through parasite-induced mortality [12].

Species of *Microphallus* can manipulate host circadian rhythmicity, increasing their activity during periods when predators are more active and thereby enhancing transmission [58]. Similarly, the acanthocephalan *Profilicollis altmani*, reported as the main parasite of *Emerita* (Table 1), can alter the burrowing time, escape speed, and carapace coloration of their host, facilitating predation [59,60]. Infection may also reduce host metabolic rate and divert energy from growth, reproduction, and locomotion, as reported for *Emerita analoga* [61].

In both parasite groups, the infective larval stages develop within *Emerita*, specifically as cystacanths for acanthocephalans and metacercariae for trematodes, which are targets of the host immune response. Constant immune activation requires energy output, and hosts may downregulate their metabolism under persistent infection to compensate for this demand [62]. In larger *Emerita* specimens, higher proportions of melanized trematode cysts (an indicator of immune response) have been observed, suggesting that older individuals have a more effective immune response to parasitic infections [12]. Parasite presence and intensity are influenced by environmental sanitary conditions (e.g., levels of fecal coliforms and domestic sewage discharge), organic matter content, and the presence of definitive hosts, making parasite load a potential indicator of beach health [63].

Except for *Proleptus carvajali*, a nematode that parasitizes coastal elasmobranchs, most parasites reported in *Emerita* use coastal birds as definitive hosts. According to Torres et al. [63], *P. carvajali* was absent in ‘unhealthy’ beaches, suggesting that nematodes are more sensitive to eutrophication. Prey availability and environmental quality drive coastal bird abundance and parasite release [64,65,66,67,68,69], while heavily infected individuals are more susceptible to predation, supporting the extended phenotype concept, in which parasite genes manipulate host behavior for their own benefit [12,47,70].

The presence of coastal birds strongly influences the prevalence and intensity of *Profilicollis* and *Microphallus* species [51], and climate-driven variation in bird distributions among localities [71,72] can alter where parasites are released into the environment. The mobility of definitive hosts drives the parasite’s distribution, whereas intermediate hosts, such as the *Emerita* species, have limited benthic mobility. As a result, parasite transmission in coastal ecosystems emerges from the interplay between biotic and abiotic factors, which act as ecological filters structuring host–parasite interactions across spatial scales [51,73].

Although evolutionary transitions toward parasitism are rare, the bivalve *Kurtiella pedroana* (Lasaeidae) may represent an intermediate case. This species is typically an ectocommensal (epibiotic); however, some individuals were found living internally within the hemocoel, lacking byssal threads and potentially feeding on hemolymph, as parasites would. These internal individuals are smaller and likely unable to exist, suggesting that this endoparasitic strategy may represent a dead end. Nevertheless, the facultative parasitism observed points to a possible evolutionary pathway to parasitism [74].

#### 2.2.3. Epibiosis

Epibiosis is defined as a facultative, non-parasitic interaction in which one organism (the epibiont) attaches to the body surface of another (the basibiont) [75]. There is controversy in the literature over whether epibiosis represents a truly commensal interaction (without harm to the basibiont), a mutualistic interaction (with benefits for both species), or even whether it often imposes costs that verge on antagonism [76]. While epibionts may offer their basibionts associational resistance against predation [77,78], other studies have demonstrated that epibionts can induce behavioral changes, reduce growth rates, diminish foraging capacity, and increase sensitivity to contaminants [79,80].

In *Emerita*, epibiont organisms typically attach to the external portion of the carapace, where they live [75,81]. Epibiosis was identified in 15 separate records within our synthesis, and the studies mainly describe epibiosis involving algae and mussel fouling [5,51,81,82]. All documented cases of epibiosis have involved the southern population of *E. analoga* along the Pacific coast of the Americas, a region influenced by upwelling events [83] and the cold, nutrient-rich Humboldt Current (or Peru Current) [84]. The high primary productivity and abundant availability of epibiont propagules in these areas [84] may explain the higher occurrence of epibiosis in *E. analoga* compared to other species in the genus, in addition to *E. analoga* being a comparatively more-studied species than the others [14].

Firstater et al. [5] indicated that the presence of the green algae *Enteromorpha* spp. (=*Ulva* spp.) reduced burrowing speed and may have facilitated the presence of other encrusting organisms beneath its canopy, including algae as well as the crustacea barnacle *Balanus laevis* and the annelida polychaete *Phragmatopoma moerchi*. Lower reproductive investment was also recorded in the presence of green algae (*Ulva intestinalis* and *U. lactuca*), brown algae (*Ectocarpus* spp.), and red algae (*Polysiphonia* spp.) [85]. Fouling by *Ulva* spp. may also increase vulnerability to predation by hindering escape behavior, camouflage, and burrowing efficiency [81]. Thus, like parasitism, epibiosis can influence predation, reproductive performance, and other key processes regulating population dynamics. However, the impact of these effects is poorly studied.

#### 2.2.4. Competition

Competition involving *Emerita* was identified in 3 separate records within our synthesis, focusing on both interspecific and intraspecific dynamics. Despite the physically stressful conditions of sandy beaches, these records provide quantitative and qualitative evidence that competition influences intertidal distribution and abundance [86,87,88].

In sandy beach ecosystems, competitive interactions can be significant even under physically stressful conditions, influencing the distribution and abundance of macroinfaunal species in the intertidal zone [8]. Both intraspecific competition (between juveniles and adults) and interspecific competition can shape zonation patterns and size segregation [8,89,90,91,92]. Direct interference, such as the displacement of individuals during burrowing, may increase exposure to the swash or predators, negatively affecting feeding, migration, and survival [10,92]. Density-dependent processes have been suggested to play a more important role than physical factors in regulating the population dynamics of *Donax hanleyanus*, which acts as a regulatory force among suspension-feeding species [93,94].

Studies have documented competitive interactions between *Emerita* species and other macrofaunal taxa occupying similar ecological niches. The reports include *E. analoga* on Chilean beaches (competing with *Donax variabilis*) [8], *E. brasiliensis* on Brazilian beaches (with *D. hanleyanus*) [92], and *E. talpoida* on U.S. beaches shared with *Mesodesma donacium* [95]. Significant negative correlations between the densities of these three species of *Emerita* and co-occurring bivalves were observed, suggesting direct competition [8,95,96].

Direct interference, including the physical displacement of bivalves or other crabs during burrowing, further contributes to spatial segregation [8,95]. The strength of competitive effects often depends on body size, substrate type, tidal stage, and species density, and may drive co-occurrence avoidance, niche partitioning, and zonation patterns within intertidal communities [8,92].

Intraspecific competition involves sexual competition and interactions among individuals with differing competitive abilities [97,98]. Among decapod crustaceans, such intraspecific competition can influence growth, maturity, injury rates, survival, and final carapace size, ultimately affecting ecological success [99]. In *Emerita*, intraspecific competition has been reported for *E. brasiliensis* in Brazil and Uruguay, with asymmetric competition between sexes leading to unequal access to resources and a disadvantage for males, contributing to the predominance of females on temperate beaches and more balanced sex ratios on subtropical beaches [100]. Evidence also suggests competition for space between juveniles and adults in *Donax* spp., within the intertidal zone [89,90,91]. Although zonation patterns by shore height have been observed among *Emerita* species, they have generally been attributed to individual adjustments to enhance survival rather than to intraspecific competition [96,101,102,103].

#### 2.2.5. Symbiosis

Symbiotic interactions were identified in 3 separate records within our synthesis, encompassing both interspecific associations with microorganisms and intraspecific male-female aggregations. Qualitative and quantitative evidence regarding interspecific symbiosis is focused on the ichthyosporean *Enterobryus halophilus* in *E. portoricensis* and *E. talpoida* [104,105]. According to these studies, although spores with no identifiable function or effect were observed in *E. talpoida*, the relationship cannot be defined as parasitic because there is no clear evidence that *E. halophilus* harms the mole crab. In addition, the prevalence and intensity of *Enterobryus halophilus* in *E. talpoida* do not vary with host sex; instead, its populations follow host population dynamics, exhibiting seasonal variations in its presence [105].

## 3. New Insights into *Emerita brasiliensis* and *Emerita rathbunae*

### 3.1. Study Area

**Figure 4:**
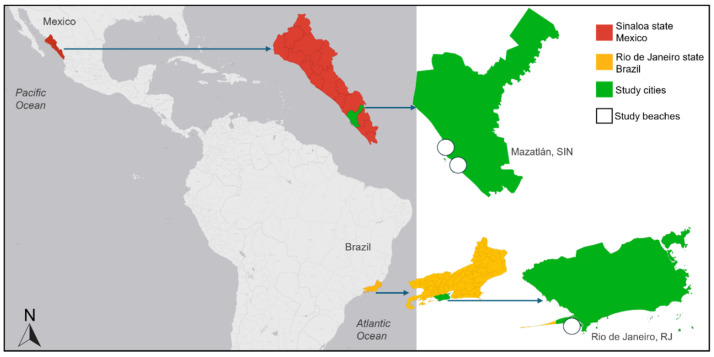
Sampling regions. The state of Sinaloa (Mexico, **top**) is shown in red, while the state of Rio de Janeiro (Brazil, **bottom**) is shown in yellow. The cities where sampling occurred (Mazatlán and Rio de Janeiro) are shown in green, with white circles demonstrating the approximate sampling location.

The study sites were selected to broaden the geographical and taxonomic scope of *Emerita* ecological interactions, covering two distinct oceanographic provinces: the Western Atlantic (Brazil) and the Eastern Pacific (Mexico) (Figure 4).

In Brazil (South Atlantic), sampling was conducted at Marambaia Beach (23°3′16.96″ S, 43°34′50.50″ W), a 48 km sandy barrier spit in Rio de Janeiro. This area is a legally protected ecological sanctuary (Marambaia Area of Special Environmental Interest) with restricted human access, preserving a stable environment for studying the natural interactions of *E. brasiliensis* and its native predators.

In Mexico (Eastern Pacific), two beaches with distinct shore configurations were sampled in Mazatlán (Gulf of California): Isla de la Piedra (23°11′3.69″ N, 106°23′59.96″ W), a 16 km semi-isolated peninsula managed as a sustainable tourism zone, and Gaviotas Beach (23°14′24.9″ N, 106°27′1.44″ W), a 2 km urban beach within Mazatlán’s Golden Zone.

The inclusion of these diverse coastal settings in both countries enables a more comprehensive understanding of how ecological interactions manifest across the genus’s tropical distribution.

### 3.2. Sampling Methods and Analysis

Populations of *E. brasiliensis* were monitored at Marambaia Beach (Brazil) from March 2022 to March 2025. For *E. rathbunae*, monitoring was conducted at Isla de la Piedra and Gaviotas beaches (Mexico) from May to October 2024. At all sites, individuals were collected using a species-directed sampling approach, targeting aggregated patches within the swash zone until a minimum sample size of n = 60 individuals per event was reached. This method ensured the representation of various size classes and life stages.

In the laboratory, specimens were sexed (male, female, or ovigerous female) and measured for carapace length (CL) using digital calipers. Each individual was meticulously inspected under a stereomicroscope to record the presence, prevalence, and intensity of epibiosis, parasitism, and intraspecific symbiosis. The identification of epibionts and parasites is described in detail below.

### 3.3. New Interactions Observed for Emerita brasiliensis

#### 3.3.1. Epibiosis (First Records for the Species)

As mentioned above, epibiosis by macroalgae has previously been documented for *Emerita* spp. [5,81,85], but in the case of *E. brasiliensis*, this relationship has remained unpublished until now. In March 2025, during sampling, epibiosis by macroalgae was observed on two non-ovigerous females of *E. brasiliensis* (A and B). The specimens A and B were collected, placed alive in containers with sand and seawater, and transported to the laboratory. Neither individual was in a molting stage (shedding of the old exoskeleton). The two epibiotic macroalgae species were visibly different.

**Figure 5:**
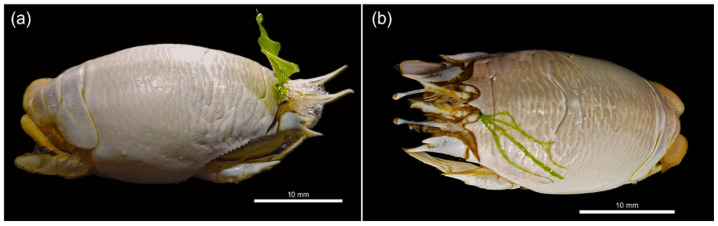
Individuals of *Emerita brasiliensis* with the epibiotic macroalgae *Ulva lactuca* (**a**) and *Ulva* cf. *linza*; (**b**) attached to the frontal part of the carapace.

Individual A of *E. brasiliensis* measured 22 mm (cephalothorax length, CL), and the epibiont species was identified as *Ulva lactuca* (Figure 5a). The identification was based on morphological characteristics, including thallus shape (foliose, laminar, and broad) and texture (broad blade), as well as the site of occurrence, given that this species is widely distributed in intertidal regions of southeastern Brazil [106,107].

Individual B measured 20 mm in CL, and the epibiont species was identified as *Ulva* cf. *linza* (Figure 5b). Its identification was based on morphological characteristics, including thallus shape (narrow, long, and ribbon-like) and texture (rigid and flexible), as well as the site of occurrence, as this species typically inhabits intertidal zones with rough waters and exposed areas [108,109]. However, it is rare and poorly documented in Brazil.

For both individuals A and B, the epibiotic macroalgae were attached beneath the carapace, on the frontal area near the rostrum, and laterally close to the base of the eye stalks and the filtering antennae. Given the attachment location, it can be inferred that the arrival of the epibiotic algae occurred in their sporophytic or gametophytic phase during the crab’s filter-feeding activity, initiating development as epibionts at that site. Both individuals A and B were adults and close to their maximum size (26 mm) [96], and, based on the size of the algae, it is suggested that attachment occurred during the last molting phase before capture, as also proposed by Williams [110] for *E. talpoida* with attachment of *Enteromorpha flexuosa*.

Species of *Ulva* have a remarkable ability to colonize both inert and biological substrates in shallow, nutrient-rich, low-energy marine environments [111,112] and the adhesion of *Ulva* begins with the settlement of motile zoospores, which use their flagellated anterior pole to detect and attach to suitable surfaces, secreting adhesive glycoproteins that ensure firm fixation, followed by germination and rhizoid development [113]. This finding expands the known spectrum of *Ulva*’s epibiotic substrates—which includes bivalves (*Perna perna*, *Anomalocardia flexuosa*), crabs (*Callinectes* spp.), and even other macroalgae (e. g., *Sargassum*, *Gracilaria*)—and highlights its ecological versatility and opportunistic colonization strategy under eutrophic coastal conditions.

#### 3.3.2. Parasitism

Individuals larger than 3 mm in CL collected at Marambaia from March to December 2022 were dissected under a stereomicroscope to detect parasite occurrence. Cystacanths, located mainly in the host’s hemocoel and intestine, were carefully removed and identified as *Profilicollis altmani* based on their morphology and compared with specimens molecularly identified by Cabrini et al. [54] from Rio de Janeiro.

A total of 1159 specimens of *E. brasiliensis* were examined, of which 141 individuals were infected, resulting in a prevalence of 12.16% (95% Confidence Interval, 10.2–14.1%). These individuals had at least one cystacanth lodged in their bodies, indicating that parasitosis occurs at a consistent level and is regularly present within the host population, rather than being an occasional occurrence. The intensity of the parasitosis ranged from one to three cystacanths per host, but only the large females (≥18.52 mm in CL) harbored more than one cystacanth. The minimum body size at which parasitism was detected was 10.12 mm in CL (males). A weak but significant positive correlation (Spearman’s ρ = 0.299, *p* < 0.001) revealed that larger individuals may carry higher parasite loads (Table 2 and Figure S1).

A combined generalized linear model for sex, body size, climate, and season revealed that the interaction between sex and body size was the most significant, with larger females having a higher probability of parasitism (AIC = 729.72). Female mole crabs are larger than males, and this size difference may explain the sex-related pattern in parasitism, since the infection probability is directly influenced by body size [18].

**Figure 6:**
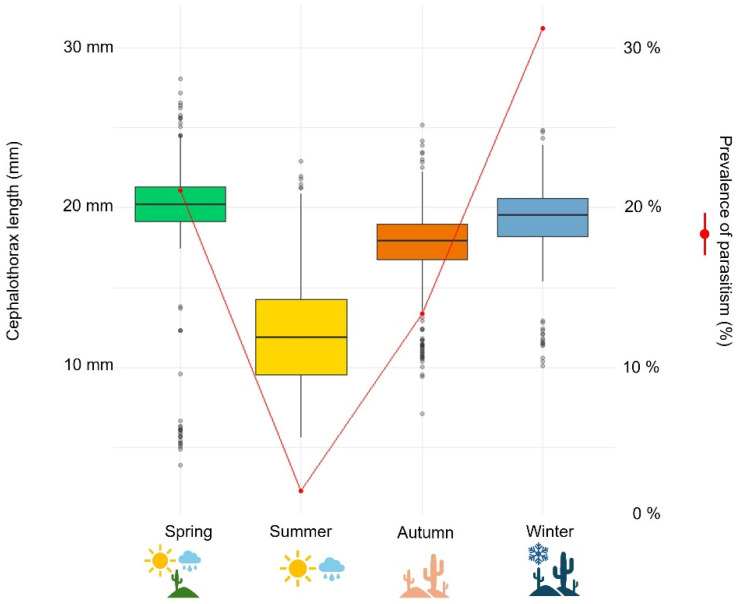
Cephalothorax length of *Emerita brasiliensis* and parasitism prevalence of *Profilicollis altimani* by season. Colors represent seasons: green (Spring), yellow (Summer), orange (Autumn), and blue (Winter). Dots represent outliers.

Larger mean body sizes of *E. brasiliensis* were observed in winter and spring (mean CL = 19 mm). These individuals also presented higher infection prevalence (31% and 21%, respectively (Figure 6). Three parasites per host were observed in two relatively large females in winter (22.2 and 20.94 mm in CL). October was the month with the largest overall mean body size (19.8 mm) and the highest parasite prevalence (29.55% of individuals infected).

### 3.4. Interactions Observed for Emerita rathbunae

#### 3.4.1. Epibiosis (Novel for the Species)

**Figure 7:**
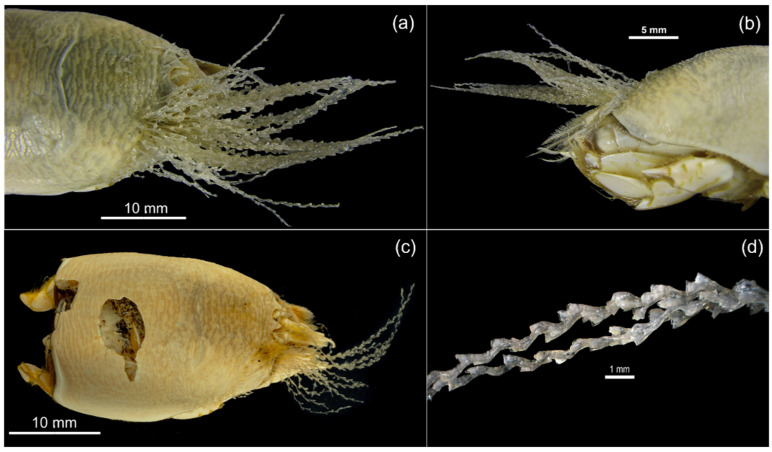
Individual of *Emerita rathbunae*, damaged during sampling, with the epibiotic hydrozoan *Eucheilota bakeri* attached. (**a**,**c**) dorsal view; (**b**) lateral view; (**d**) detail of the hydrozoan.

On Isla de la Piedra’s beach, a novel epibiotic interaction of *E. rathbunae* with a colonial hydrozoan was observed during fieldwork (Figure 7). The specimen, a non-ovigerous female (28 mm in CL), was fixed in ethanol and transported to the laboratory. Because the epibiont was located on the carapace, attachment should occur after the crab’s last molt, when the carapace size approaches its maximum known CL [114] of 36.39 mm for non-ovigerous females of this species. Morphological analysis identified the epibiont as the hydrozoan *Eucheilota bakeri* (Lovenellidae) based on comparisons with original descriptions and subsequent literature [115,116,117].

*Eucheilota bakeri* is a common hydrozoan inhabiting shallow coastal waters of the Eastern Pacific, particularly in southern California and Mexico [118,119]. Its life cycle alternates between a sessile benthic colonial stage (polyp/hydroid) and a free-living planktonic stage (medusa).

The species is epizoic on living bivalves inhabiting lower intertidal to subtidal sand beaches, forming tufts up to 20 mm high, typically on *Donax* spp., but associations with *Tivela* spp. and other hydroids have also been reported [117,120]. In the study region, *E. bakeri* was previously observed on *Donax punctatostriatus* in 1979 and 2014 (unpublished data). Its association with bivalves has been suggested to be seasonal: few colonies are seen in winter, but nearly all clams host hydroids in summer [121].

#### 3.4.2. Multiple Male-Female Attachment (First Records for the Species)

**Figure 8:**
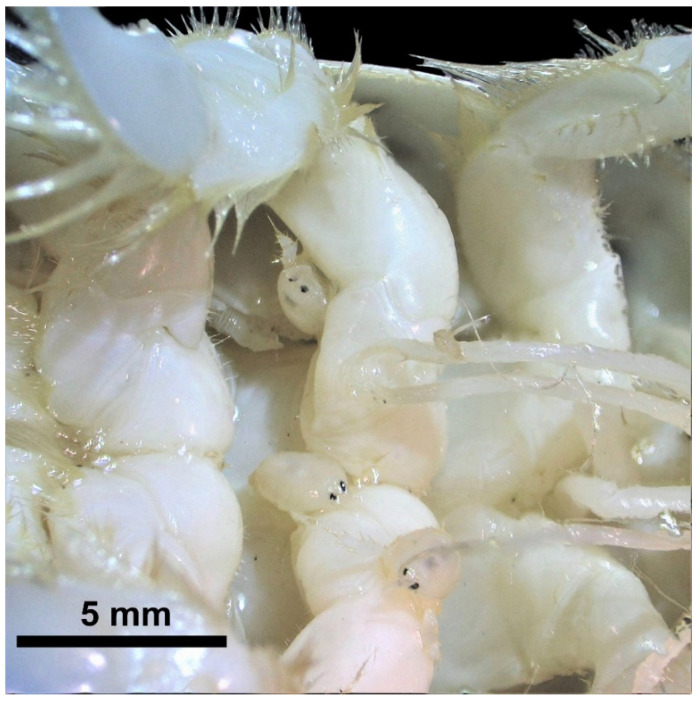
Three symbiotic males (~2 mm) attached to the third pair of pereopods, adjacent to the gonopore, of a non-ovigerous *Emerita rathbunae* female.

In June 2024, an unprecedented intraspecific interaction was observed, characterized by multiple males firmly attached to the ventral surface, adjacent to the gonopore, on the third pair of pereopods of a single adult female *E. rathbunae* from Gaviotas beach (Figure 8). Each male displayed conspicuous, well-developed gonopods (copulatory pleopods). Yet, their carapace lengths (<3 mm) were much smaller than the 10–12 mm average of free-living males in the same population during the same sampling period. According to Efford [122]. This indicates a precocious sexual development (neoteny) in males. Carapace lengths for *E. rathbunae* males range from 2.22 to 14.18 mm [114].

The three males, measuring 2.04, 2.06, and 2.15 mm in CL (Figure 8), lacked developed ocular peduncles, which generally indicates recent recruitment into the population [96]; however, they possessed a fully formed fifth pair of pereopods, a feature characteristic of mature males of this species [56,114]. Observations under the stereomicroscope revealed that the males clung by the dactyls of their fourth pereopods as precisely described by MacGinitie [123] for *E. talpoida*, who reported the ease with which the males attach to females.

This symbiotic mating strategy, while rare within the genus, may enhance reproductive success in the highly turbulent intertidal zone, where chemical cues would disperse too rapidly for pheromonal attraction and aggregation behavior that would normally bring individuals into close contact [123,124]. In a strategy aimed at maximizing reproductive success, *E. asiatica* exhibited a peculiar mating behavior that was described as heterosexual rape [123]. In this phenomenon, tiny males indiscriminately deposit spermatophores on immature females without any apparent pheromonal attraction guiding their choice. During copulation, numerous minute males cling to the ventral surface of the female’s abdomen and affix their sticky spermatophore masses onto her pleopodal region. Although neotenic males may attach to much larger non-ovulatory females, spermatophore deposition occurs only when females enter the pre-ovulatory period [122].

Few population studies have focused on *E. rathbunae* [19,114,125]. However, symbiotic males were reported at the same locality in a previous study [114]. In this genus, males generally exhibit neoteny, characterized by precocious sexual maturity and a slower rate of body development than in females, and are smaller than females [122]. For some *Emerita* species (*E. emeritus*, *E. talpoida*, and *E. rathbunae*), the males live attached to females on the ventral side near the sexual pore, establishing an intraspecific symbiotic interaction [56,101,114].

Subramoniam [56] observed tiny males of *E. asiatica* (=*E. emeritus*) attached to or wandering on the ventral surface of females for prolonged periods. This behavior has been interpreted as sexual coercion, since neotenic males attach to young females and deposit spermatophores without the pheromonal cues that normally trigger female receptivity [56,124,126]. This behavior may represent a strategy to maximize reproductive success. The male-female aggregation was observed in *E. analoga* [123,127], which exhibited two seasonal peaks of male-female aggregation—early spring and late summer—during the highest reproductive period for females [127]. Subramoniam [56] suggested that males have a commensal relationship with females that may be semi-parasitic, given their direct dependence on females, as indicated for *E*. *talpoida* by Wharton [128]. However, these suggestions were not fully supported, as no direct evidence of any harm to the female hosts was provided. Similarly, no deleterious effects have been observed in *E. rathbunae* from the Mexican, although 5% of the males were found living attached to females [114].

#### 3.4.3. Parasitism and Multiparasitism (Novel for the Species)

In October 2024, an active search was conducted at Isla de la Piedra beach to collect *E. rathbunae* specimens to identify their parasite species and assess their prevalence and intensity. Thirty-four individuals (CL > 10 mm) were collected by hand, transported alive to the laboratory, and placed in containers with sand and seawater for taxonomic identification of their parasites. During preliminary examinations, two types of parasites were detected: acanthocephalan cystacanths and platyhelminth metacercariae. In several cases, both parasites were found in the same specimen, indicating multiparasitism with simultaneous infection.

**Figure 9:**
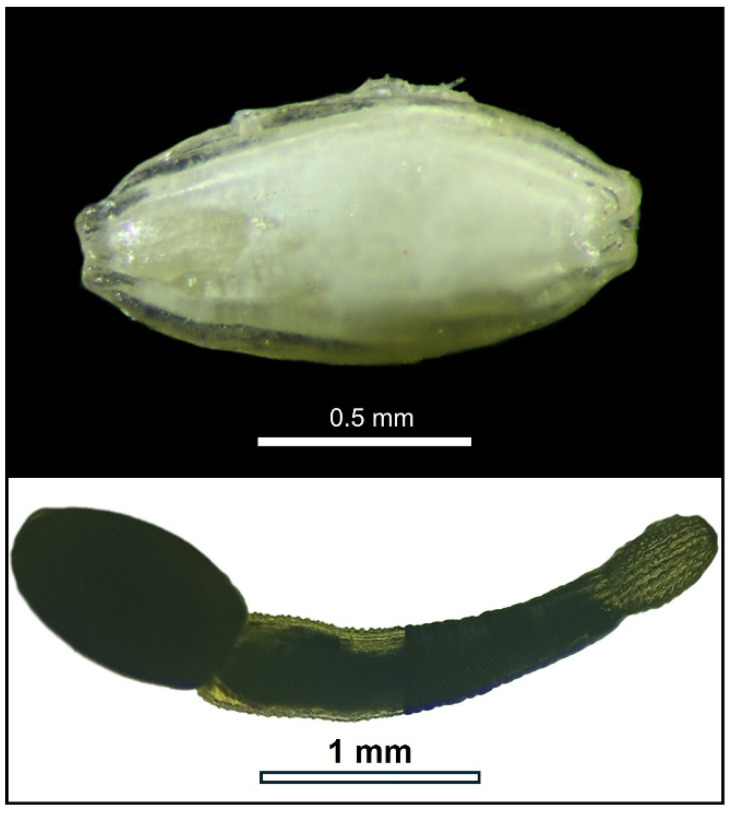
*Profilicollis altmani* cystacanth before (**top**) and after (**bottom**) proboscis eversion following 10–15 min in distilled water and 1 h in the fridge to facilitate the eversion of the proboscis.

**Figure 10:**
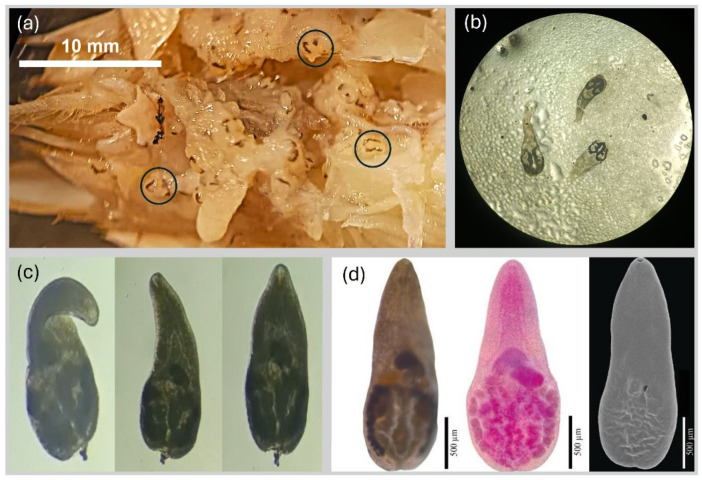
(**a**) *Maritrema* sp. encysted under the host carapace (encysted metacercariae circled); (**b**) excysted individuals after 10–15 min in physiological saline solution; (**c**) sequence of movements captured on video following excystment; (**d**) specimens prepared using three different visualization techniques (left: whole mount under light microscopy, center: specimen stained for histological visualization, right: scanning electron microscopy (SEM) image highlighting surface morphology).

In the laboratory, hosts were measured and sexed. All specimens were females or ovigerous females (47%), likely because the collection method favored the largest individuals. After removing the carapace, *Emerita* specimens were observed under a stereomicroscope. Cystacanths and metacercariae located in the abdominal cavity and hemocoel were carefully extracted using fine forceps and placed in 0.9% saline solution to promote relaxation. After 10–15 min, acanthocephalans everted their proboscis (Figure 9), while the cystic membrane of metacercariae was gently ruptured with a needle to release the individual for identification (Figure 10, Video S1). All parasites were counted and classified as acanthocephalans or platyhelminths, and everted (acanthocephalan) or excysted (platyhelminth) specimens were fixed for subsequent morphological and histological analysis.

Parasites were identified based on comparative morphological descriptions. Specimens initially identified as *Maritrema* sp. were molecularly validated using partial sequences of the nuclear 28S large subunit (LSU) ribosomal RNA gene (D1–D3 domains). Genomic DNA was extracted from ethanol-fixed specimens, and the target region was amplified by PCR and sequenced. The resulting sequences were analyzed using the BLASTn tool (2.17.0, NCBI) to confirm taxonomic identity at the genus level.

Identification of parasites revealed the acanthocephalan as *Profilicollis altmani* (Acanthocephala: Polymorphidae), consistent with morphological comparative descriptions [54]. The platyhelminth was identified as *Maritrema* sp. (Digenea: Microphallidae) based on morphological and molecular similarities, using diagnostic features (see [129]). The presence of metacercariae of *Maritrema* represents the first record of this genus in *Emerita* from Mexican coasts. In the Neotropical region of Mexico, a few species have been described as associated with aquatic birds, including *M. kostadinovae* [130,131,132]. Phylogenetic analysis based on nuclear LSU (Figure S2) indicates that the specimens recovered in this study are closely related to *M. kostadinovae* and may represent a new species.

**Figure 11:**
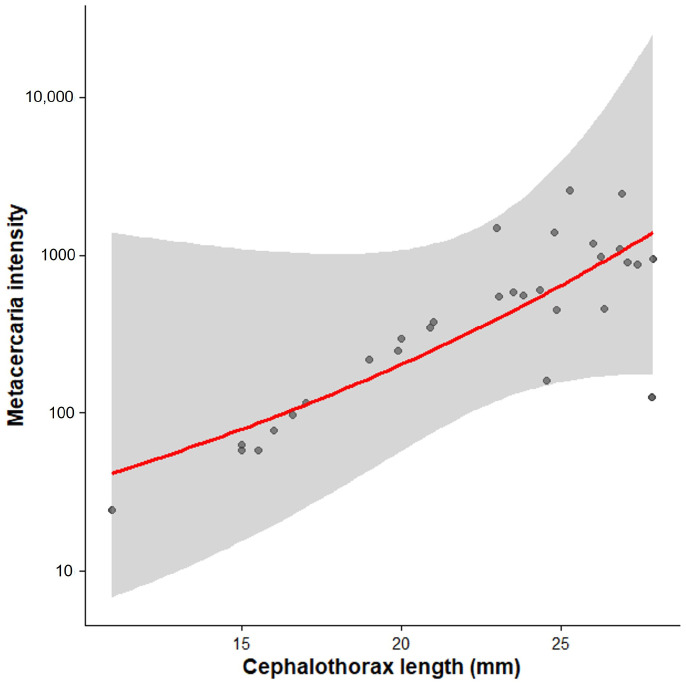
Exponential increase in metacercaria (*Maritrema* sp.) intensity with host body size (cephalothorax length) in *Emerita rathbunae*. The red curve shows the fitted Negative Binomial Generalized Linear Model (log link). The *y*-axis is on a logarithmic scale. The shaded area indicates the 95% confidence interval. Black circles indicate individual observations.

The prevalence of *Maritrema* sp. was 100% in the collected specimens, with infection intensity ranging from 24 to 2600 metacercariae per host. *Profilicollis altmani* was found in 7 individuals (23%), each with a single cystacanth. For *Maritrema* sp., a strong positive correlation was found between host size and infection intensity (Spearman’s ρ = 0.813, *p* < 0.001), indicating that larger individuals harbor more parasites. A generalized linear model (GLM) with a negative binomial distribution showed that parasite intensity increased exponentially with host length (β = 0.245 ± 0.024 SE; z = 10.29; *p* < 0.0001), indicating that each 1 mm increase in host length results in an average increase of approximately 28% in parasite load (Figure 11). Neither host sex nor size showed a significant association with the occurrence of *P. altimani* (Fisher’s exact test, *p* = 0.693 and *p* = 0.804, respectively), likely due to the small sample size.

Parasitism is the main ecological interaction reported for *E. rathbunae* [12,18]. Infections by *M. nicolli*, another Microphallidae species from the Mexican Pacific, can reach up to 6000 parasites per host [12]. Parasites are known to cause mortality in marine crustaceans under natural conditions [6,12,62,132]. Decreases in mean parasite intensity among larger crabs have been linked to the death of heavily infected individuals [133,134]. In this context, higher-frequency monitoring would be required to assess potential mortality effects among larger *E. rathbunae* individuals.

## 4. Conclusions

This study provides a comprehensive, data-driven synthesis of the ecological role of *Emerita* species, integrating a review of the global literature with novel empirical evidence from the South Atlantic and the Tropical Pacific. Our findings indicate that predation and parasitism are strong drivers of *Emerita* population dynamics globally. Empirically, we provide the first evidence of epibiosis in *E. brasiliensis* (with *Ulva* spp. and *Eucheilota bakeri*), challenging the previous paradigm that such interactions are geographically restricted to the Humboldt Current region. Furthermore, the quantitative parasitological data from *E. brasiliensis* and *E. rathbunae* corroborate global trends where host size and sex (females) are the main determinants of parasite intensity. The identification of *Maritrema* in *E. rathbunae* represents an unprecedented taxonomic record, expanding the known parasitic diversity for the genus. Finally, the striking similarities between the ecological threats and interactions observed in Mexico and Brazil suggest that *Emerita* species function as universal sentinels for sandy beach integrity. Within this context, we suggest that while a healthy environment provides the necessary conditions for these complex biotic links to persist, the persistence of such interactions may, in turn, serve as an indicator of ecosystem functional stability. A healthy environment is a prerequisite for maintaining these complex links, including stable parasite–host dynamics that depend on the presence of final hosts. Monitoring these biological interactions thus provides a robust indicator of environmental conditions. This integrative approach is essential for developing transregional conservation strategies for these vital yet vulnerable coastal ecosystems.

## Figures and Tables

**Table 1:** Global ecological interactions of *Emerita* species organized by interaction type, interaction genera or species, and their taxa.

Interaction Type	Interacting Genera or Species	Taxon
Competition	*Donax hanleyanus*, *D. variabilis*	Mollusca
Competition	*Mesodesma donacium*	Mollusca
Epibiosis	*Ectocarpus* spp.	Brown algae
Epibiosis	*Mytilus californianus*	Mollusca
Epibiosis	*Polisiphonia* spp.	Red algae
Epibiosis	*Phragmatopoma moerchi*	Annelida
Epibiosis	*Balanus laevis*	Crustacea
Epibiosis	*Ulva flexuosa*, *U. intestinalis*, *U. lactuca*	Green algae
Epibiosis	*Semimytilus algosus*, *S. patagonicus*	Mollusca
Epibiosis	*Chondracanthus chamissoi*	Red algae
Parasitism	*Profilicollis altmani*	Acanthocephalan
Parasitism	*Kurtiella pedroana*	Mollusca
Parasitism	*Proleptus carvajali*	Nematoda
Parasitism	*Microphallus nicolli*	Platyhelminthes
Parasitism	*Spelotrema nicolli*	Platyhelminthes
Parasitism	Trypanorhyncha (order)	Platyhelminthes
Predation	*Hemipodus olivieri*	Annelida
Predation	*Anas platyrhynchos*	Aves
Predation	*Arenaria interpres*	Aves
Predation	*Calidris alba*	Aves
Predation	*Charadrius alexandrinus*	Aves
Predation	*Chroicocephalus maculipennis*	Aves
Predation	*Haematopus palliatus*	Aves
Predation	*Larus modestus*	Aves
Predation	*Numenius phaeopus*	Aves
Predation	*Pluvialis squatarola*	Aves
Predation	*Tringa nebularia*	Aves
Predation	*Xenus cinereus*	Aves
Predation	*Arenaeus cribrarius*	Crustacea
Predation	*Ocypode quadrata*	Crustacea
Predation	*Ovalipes ocellatus*, *Ovalipes* sp.	Crustacea
Predation	*Anchoa hepsetus*	Fish
Predation	*Arius felis*	Fish
Predation	*Leiostomus xanthurus*	Fish
Predation	*Menidia menidia*	Fish
Predation	*Menticirrhus littoralis*, *M. saxatilis*	Fish
Predation	*Pomatomus saltatrix*	Fish
Predation	*Trachinotus carolinus*, *T. godei*	Fish
Predation	*Umbrina coroides*	Fish
Predation	*Olivancillaria vesica*, *O. auricularia*	Mollusca
Symbiosis	*Enterobryus halophilus*	Protozoa

**Table 2:** Mean size of cephalothorax (mm), percentage of *Emerita brasiliensis* individuals parasitized 609 by *Profilicollis altmani* from March to December 2022 (** indicates months in which more than one parasite per host was recorded), percentage of prevalence by sex for each month (F: female, OF: ovigerous female, and M: male); bold highlights indicate values above the 10%.

Month	Mean Size of Cephalothorax	Sample Sizes	Infected Individuals (%)	F (%)	OF (%)	M (%)
**March**	12.11 mm	431	2.31	4.50	0.00	0.00
**April**	16.99 mm	42	4.76	5.56	0.00	0.00
**May**	17.05 mm	117	9.40	**11.54**	8.22	**11.11**
**June**	18.07 mm	125	**20.00**	**26.00**	**16.18**	**16.67**
**July**	18.31 mm	73	34.25 **	**25.00**	**33.93**	**38.46**
**August**	19.75 mm	68	27.94 **	**47.06**	**22.92**	0.00
**September**	16.60 mm	14	21.43 **	0.00	**25.00**	**25.00**
**October**	19.87 mm	88	29.55 **	**21.05**	**34.92**	0.00
**November**	19.12 mm	126	15.08 **	8.82	**20.51**	0.00
**December**	5.30 mm	75	1.33	0.00	**50.00**	0.00
**Total**	16.31 mm	1159	**12.16**	**10.48**	**21.78**	2.69

## Data Availability

Data are available upon request.

## Supplementary Materials

The following supporting information can be downloaded at https://www.mdpi.com/article/10.3390/biology15040311/s1, Figure S1: Probability of *Emerita brasiliensis* being parasitized. F: females; J: juveniles; M: males; O: ovigerous females.; Table S1: Ecological interaction records for species of *Emerita*. Interacting species, their taxa, and sources; Figure S2: Maximum likelihood tree and consensus Bayesian Inference trees inferred with large subunit (LSU) from nuclear DNA; numbers near internal nodes show posterior probabilities (BI) and ML bootstrap values. Scale bar = number of substitutions; dash in the nodes represent values less than 50% of bootstrap; Video S1: Live observation of *Maritrema* sp. (Digenea: Microphallidae) showing contractile movements and motility. References [135,136,137,138,139,140,141,142,143,144,145,146,147,148,149] are cited in Supplementary Materials.
