# Supplementary material for: Ecological Interactions on Sandy Beach Ecosystems: A Global Synthesis of Mole Crabs and New Insights into Emerita brasiliensis and Emerita rathbunae (Crustacea, Decapoda, Anomura, Hippidae)
Source: Biology (Basel). 2026 Feb 10;15(4):311. doi: 10.3390/biology15040311 (PMC12937717; doi:10.3390/biology15040311)
Supplement: Supplementary file 1 [file biology-15-00311-s001.zip › biology-4060209-supplementary.pdf]

Ecological interactions on sandy beach ecosystems: a global synthesis of mole crabs and new insights into *Emerita brasiliensis* and *Emerita rathbunae* (Crustacea, Decapoda, Anomura, Hippidae)

Supplementary material

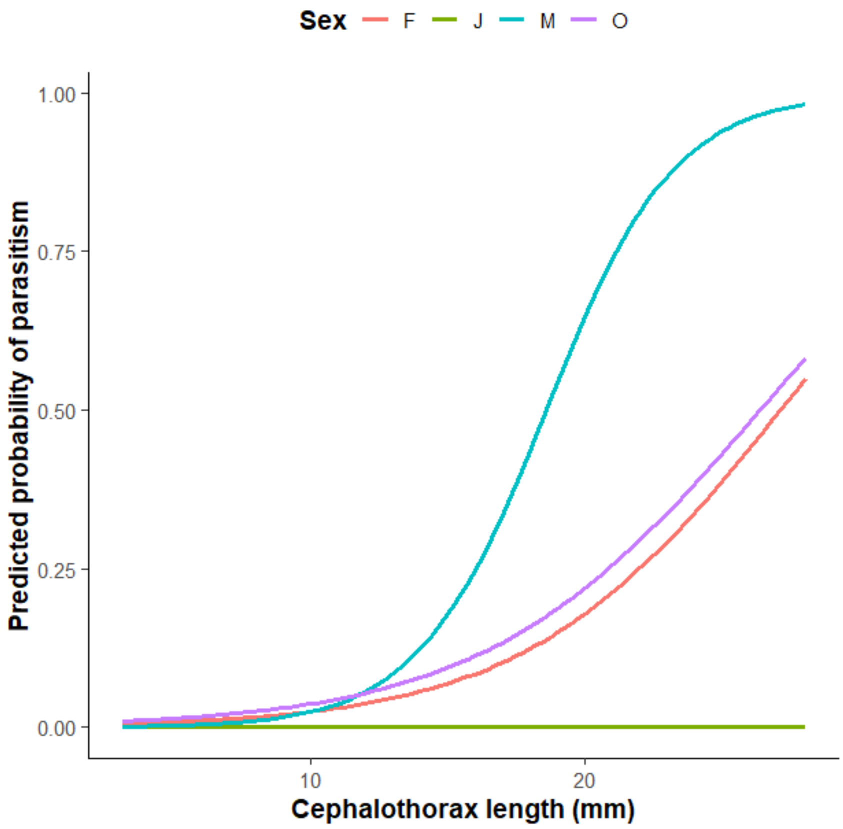

Figure S1: Probability of *Emerita brasiliensis* being parasitized. F: females; J: juveniles; M: males; O: ovigerous females.

Video S1: Live observation of *Maritrema* sp. (Digenea: Microphallidae) showing contractile movements and motility.

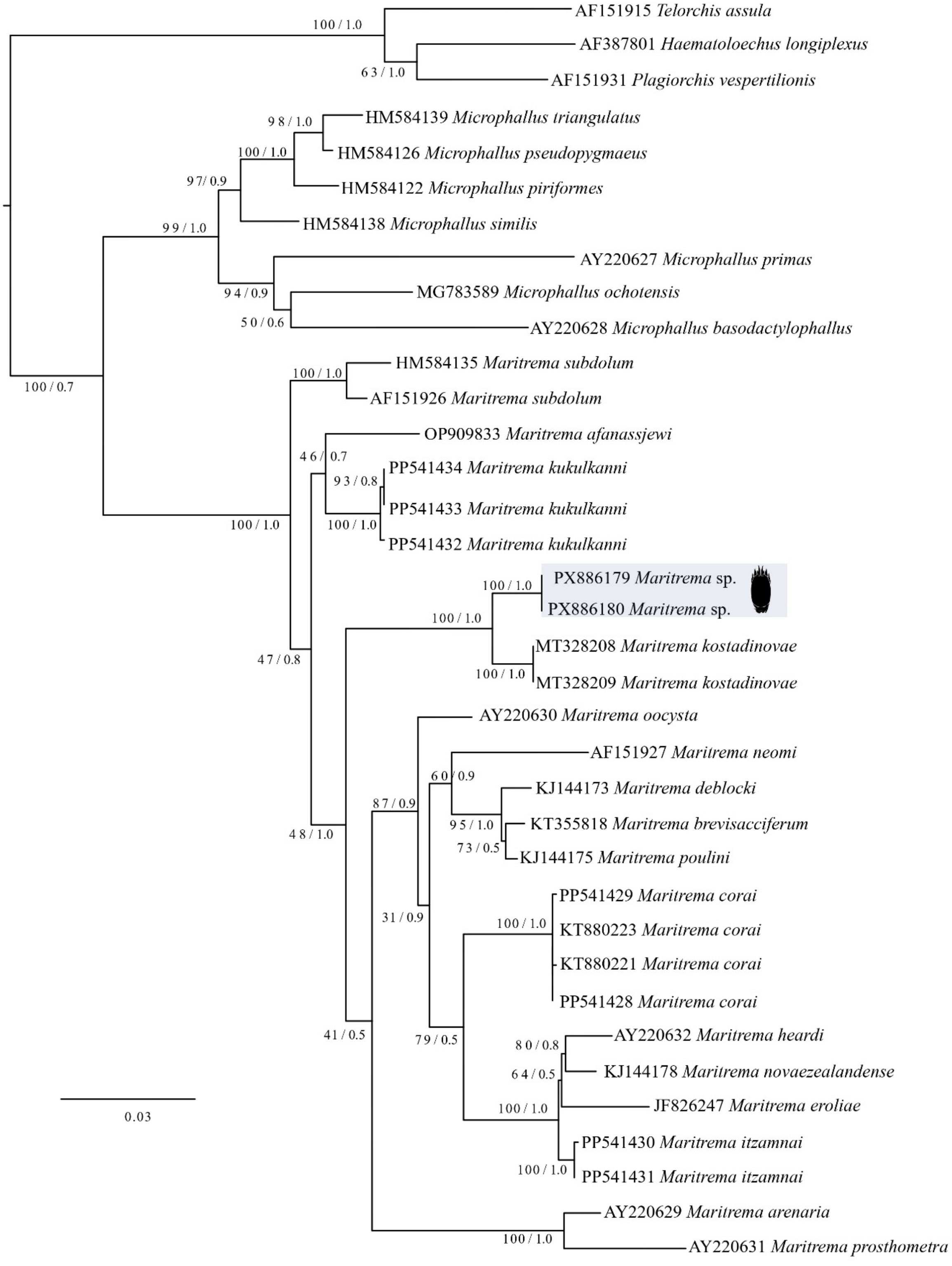

Figure S2: Maximum likelihood tree and consensus Bayesian Inference trees inferred with large subunit (LSU) from nuclear DNA; numbers near internal nodes show posterior probabilities (BI) and ML bootstrap values. Scale bar = number of substitutions; dashes in the nodes represent values less than 50% of bootstrap.

Table S1: Ecological interaction records for species of Emerita. Interacting species, their *taxa,* and sources.

Interaction type	Interacting species	Taxon	Sources
**Competition**	*Donax hanleyanus, D. variabilis*	Mollusca, Bivalvia	Cardoso & Veloso, 2003; Horwith, 2007
**Competition**	*Mesodesma donacium*	Mollusca, Bivalvia	Dugan et al., 2004
**Epibiosis**	*Ectocarpus spp.*	Brown algae	Flores, 2020
**Epibiosis**	*Mytilus californianus*	Mollusca	Bahduri et al., 2017
**Epibiosis**	*Polisiphonia* spp.	Red algae	Firstater et al., 2009; Flores, 2020
**Epibiosis**	*Phragmatopoma moerchi*	Annelida	Firstater et al., 2009
**Epibiosis**	*Balanus laevis*	Crustacea, Cirripeda	Firstater et al., 2009
**Epibiosis**	*Ulva flexuosa, U. intestinalis, U. lactuca*	Green algae	Williams, 1949; Firstater et al., 2009; Hidalgo et al., 2010; Flores, 2020
**Epibiosis**	*Semimytilus algosus, S. patagonicus*	Mollusca, Bivalvia	Villegas et al., 2006; Flores-Carpio et al., 2024
**Epibiosis**	*Chondracanthus chamissoi*	Red algae	Firstater et al., 2009
**Parasitism**	*Profilicollis altmani*	Acanthocephalan	Oliva et al., 2007; Merloy, 2009; Constancio, 2011; Kollueu et al., 2011; Kolluru et al., 2011; Goulding & Cohen, 2014; Violante-Gonzalez et al., 2015; Rodriguez et al., 2016; Rodríguez & D'Elía, 2016; Rodríguez & Valdivia, 2017; Loh, 2017; Dio Seck, 2017; Torres et al., 2018; Bahduri et al., 2018; Figueiroa et al., 2019; Bhaduri, 2020; Bhaduri et al., 2022; Amin et al., 2022; Flores-Carpio et al., 2024; Cabrini et al., 2024
**Parasitism**	*Kurtiella pedroana*	Mollusca, Bivalvia	Bhaduri et al., 2017
**Parasitism**	*Proleptus carvajali, P.* sp.	Nematoda	Smith, 2007; Violante-Gonzalez et al., 2015; Torres et al., 2018; Flores-Carpio et al., 2024
**Parasitism**	*Microphallus nicolli, M.* sp.	Platyhelminthes	Anantaraman & Subramoniam, 1976; Violante-Gonzalez et al., 2015, 2016; Bhaduri et al., 2018; Loh, 2017
**Parasitism**	*Spelotrema nicolli*	Platyhelminthes	Smith, 2007
**Parasitism**	Trypanorhyncha (order)	Platyhelminthes	Smith, 2007; Violante-Gonzalez et al., 2015
**Predation**	*Hemipodus olivieri*	Annelida, Polychaeta	Bergamino et al., 2011
**Predation**	*Anas platyrhynchos*	Aves	Lafferty et al., 2013
**Predation**	*Arenaria interpres*	Aves	Aju et al., 2019
**Predation**	*Calidris alba*	Aves	Estelle, 1991; Maia-Carneiro et al., 2013
**Predation**	*Charadrius alexandrinus*	Aves	Aju et al., 2019
**Predation**	*Chroicocephalus maculipennis*	Aves	Morales-Torres el al., 2023
**Predation**	*Haematopus palliatus*	Aves	Silva, 2021; Linhares et al., 2022

**Predation**	*Larus modestus*	Aves	Hidalgo et al., 2010
**Predation**	*Numenius phaeopus, N. phaeopus hudsonicus*	Aves	Aju et al., 2019; Morales-Torres el al., 2023
**Predation**	*Pluvialis squatarola*	Aves	Aju et al., 2019
**Predation**	*Tringa nebularia*	Aves	Aju et al., 2019
**Predation**	*Xenus cinereus*	Aves	Aju et al., 2019
**Predation**	*Arenaeus cribrarius*	Crustacea, Brachyura	McDermott, 1983
**Predation**	*Ocypode quadrata*	Crustacea, Brachyura	Fales, 1976; Morrow et al., 2014; Pinotti et al., 2014; Tewfik et al., 2016; Do Vale et al., 2022
**Predation**	*Ovalipes ocellatus, Ovalipes* sp.	Crustacea	McDermott, 1983
**Predation**	*Anchoa hepsetus*	Fish	DeLancey, 1989
**Predation**	*Arius felis*	Fish	DeLancey, 1989
**Predation**	*Leiostomus xanthurus*	Fish	McDermott, 1983
**Predation**	*Menidia menidia*	Fish	McDermott, 1983; DeLancey, 1989
**Predation**	*Menticirrhus littoralis, M. saxatilis*	Fish	McDermott, 1983; Modde & Ross, 1983; Nelson, 1986; DeLancey, 1989
**Predation**	*Pomatomus saltatrix*	Fish	McDermott, 1983
**Predation**	*Trachinotus carolinus, T. godei*	Fish	Modde & Ross, 1983; Nelson, 1986; DeLancey, 1989; Bergamino et al., 2011; Souza & Santos, 2020
**Predation**	*Umbrina coroides*	Fish	Nelson, 1986
**Predation**	*Olivancillaria vesica, O. auricular*	Mollusca, Gastropoda	Caetano, 2001; Caetano et al., 2003; Rocha-Barreira, 2002; Bergamino et al., 2011
**Symbiosis**	*Enterobryus halophilus*	Protozoa	Cronin & Johnson, 1958; Hernández Roa & Cafaro, 2012
